# Differences in Brain Activity and Body Movements Between Virtual Reality and Offline Exercise: Randomized Crossover Trial

**DOI:** 10.2196/40421

**Published:** 2023-01-05

**Authors:** Hee Jin Kim, Jea Woog Lee, Gangta Choi, Junghoon Huh, Doug Hyun Han

**Affiliations:** 1 Department of Psychiatry College of Medicine Chung-Ang University Seoul Republic of Korea; 2 Department of Information & Technology in Sport College of Sports Science Chung-Ang University Anseong-si, Gyeonggi-do Republic of Korea; 3 Department of Human Motor Behavior College of Sports Science Chung-Ang University Anseong-si, Gyeonggi-do Republic of Korea

**Keywords:** virtual reality exercise, near-infrared spectroscopy, spectroscopy, hemodynamic, blood flow, hemoglobin, brain, prefrontal cortex, orbitofrontal cortex, immersion, virtual reality, VR, exercise, range of motion, physical activity, fitness, motion, movement, randomized, calorie

## Abstract

**Background:**

Virtual reality (VR) has been suggested to be effective at enhancing physical exercises because of its immersive characteristics. However, few studies have quantitatively assessed the range of motion and brain activity during VR exercises.

**Objective:**

We hypothesized that 3D immersive VR could stimulate body movement and brain activity more effectively than standard exercises and that the increased range of motions during 3D immersive VR exercises would be associated with orbitofrontal activation.

**Methods:**

A randomized crossover trial was conducted to compare exercises with and without VR. A total of 24 healthy males performed the same motions when exercising with and without 3D immersive VR, and the recorded videos were used for motion analysis. Hemodynamic changes in the prefrontal cortex were assessed using functional near-infrared spectroscopy.

**Results:**

There were significant differences in the total angle (*z*=−2.31; *P*=.02), length (*z*=−2.78; *P*=.005), calorie consumption (*z*=−3.04; *P*=.002), and change in accumulated oxygenated hemoglobin within the right orbitofrontal cortex (*F*^1,94^=9.36; *P*=.003) between the VR and offline trials. Hemodynamic changes in the right orbitofrontal cortex were positively correlated with the total angle (*r*=0.45; *P*=.001) and length (*r*=0.38; *P*=.007) in the VR exercise; however, there was no significant correlation in the offline trial.

**Conclusions:**

The results of this study suggest that 3D immersive VR exercise effectively increases the range of motion in healthy individuals in relation to orbitofrontal activation.

**Trial Registration:**

Clinical Research Information Service KCT0008021; https://cris.nih.go.kr/cris/search/detailSearch.do/23671

## Introduction

Virtual reality (VR) is an artificially created sensory experience using digital technology, which provides users with a subjective sense of “being there” [1]. The format of VR, referring to the structure of the information displayed, can be either 2D or 3D multimedia. The main difference between the 2 formats is the level of interactivity, with 3D environments allowing changes in the user’s point of view, navigation, and interaction with objects and people. The display devices, which are the technological equipment used to visualize the formats, are classified based on the level of immersion they provide: nonimmersive, semi-immersive, and immersive. Nonimmersive systems are simple devices that use a single screen, such as a desktop PC. Semi-immersive systems, such as the Cave automatic virtual environment and the Powerwall display, provide a stereo image of an environment using a projection linked to the observer’s position. Immersive systems, such as a head-mounted display (HMD), isolate the user from external world stimuli [2]. An HMD gradually follows the users’ movements, quantifies the position and direction of articles in reality, and creates a corresponding stereoscopic view. Users perceive themselves to be situated in a virtual environment and are temporarily unable to recognize behaviors and objects in the real environment [1].

VR is expected to have enormous potential in fields such as education, training, and entertainment [3]. In particular, the application of VR technology is promising in the field of sports, as it can overcome human limitations due to technical innovations, thus allowing for the development of effective training methods [4].

VR application in sports can increase motivation, enjoyment, and physical performance [5]. This may be due to the unique characteristics of VR, such as immersion, interaction, and infinite degrees of freedom in spatial and temporal situations [6,7]. Fox and Bailenson [8] showed that physical activity could be increased through a reinforcement mechanism using immersive VR technology. Jones et al [9] demonstrated that immersion in VR exercises was positively correlated with motivation, pleasure, and enjoyment after exercises. In addition, participants can interact with objects in a stereoscopic visual space without spatial limitations, which can be manipulated in various ways, depending on individual needs [10]. Athletes can use VR exercises to learn new sports strategies and compete with their virtual partners [10]. By using the unlimited spatial and temporal boundaries of VR, injury-prone skills can be safely trained [11]. For example, VR allows ski jumps and downhill skiing in a safe environment [11].

The shortcomings of physical exercise using an immersive VR have been attributed to the use of HMDs. Exercising while wearing an HMD can be uncomfortable due to sweating and head bumps [12]. In addition, HMDs can be impractical for movement accuracy and speed. They can also be potentially dangerous (eg, when the device blocks the vision of the moving treadmill while running [12]). Furthermore, interactive VR display systems linked to various eye-movement systems can cause motion sickness [13].

VR exercises are effectively used in the rehabilitation of patients who have lost motor skills [5,14]. They are also used to increase the physical fitness of patients with chronic medical conditions, such as hemodialysis or dementia, and developmental disorders, such as intellectual disability or autism [15,16]. Most previous studies on VR exercises have been conducted on patient groups [5,14-16]. However, VR exercises are increasingly used by athletes and other sports-related professionals, as they enable individualized training with real-time feedback without coaches [11,12]. Moreover, VR exercises have recently been widely used in the public sphere to promote physical fitness [5]. When applied to healthy individuals, VR exercise can increase exercise speed, heart rate, balance, motivation, enjoyment, adherence, executive function, and neuroplasticity [17-20]. Although many studies have suggested the qualitative effectiveness of VR exercise, few have quantitatively assessed its effectiveness [12].

Previous studies have suggested that the brain regions of interest in response to VR and physical exercises are the dorsolateral prefrontal cortex (DLPFC), ventrolateral prefrontal cortex (VLPFC), and orbitofrontal cortex (OFC) [9,21,22]. Of these 3 major prefrontal cortical regions, a functional near-infrared spectroscopy (fNIRS) study showed that VR stimuli activated the OFC [21]. The OFC has been associated with emotional and behavioral regulation, decision-making, maintenance of behavioral flexibility, and processing of anticipated rewards and punishments [23]. Inappropriate functioning of the OFC can lead to disinhibition, preservation, and impulse control problems [24]. Owing to the functions of rewards and decisions, the OFC can be associated with VR-induced immersion and flow [8,25].

This study aimed to investigate whether immersive VR technology can enhance physical exercise, in terms of the range of motion and brain activity, in healthy individuals. Kinematics and resting state fNIRS methodologies were adopted to demonstrate the quantitative effectiveness of 3D immersive VR exercise. Kinematics is useful for objectifying human movements and comparing different subjects or situations, such as before and after treatment or training in various fields (eg, sports science) [26]. For example, knee and hip angles at foot strikes during running have been quantified using 2D video recording and motion analysis software [27]. Vertical jump height, frequently used as an indirect measure of muscle power in the lower extremities, was also estimated using the video method [28]. In VR rehabilitation studies, the difference in ranges of motion before and after treatment was measured to assess improvement [29]. fNIRS is a noninvasive functional brain imaging tool that measures cortical hemodynamic activity [30]. Near-infrared light penetrates the brain, and oxygenation changes in regional cerebral blood flow are recorded [31]. fNIRS has the advantages of safety, low cost, portability, tolerance to motion artifacts, and good temporal and spatial resolution, overcoming the shortcomings of conventional brain imaging modalities, such as electroencephalography and functional magnetic resonance imaging (fMRI) [32,33]. fNIRS has been used in various fields, including sports medicine, neuroscience, behavioral science, clinical studies, and brain-computer interfaces [30].

We hypothesized that 3D immersive VR exercises would be more effective than offline physical exercises in increasing the range of motion and calorie consumption during exercise. In addition, we hypothesized that the effectiveness of 3D immersive VR exercises is associated with brain activation within the OFC before and after exercise.

## Methods

### Sample Size

The sample size was calculated using G*power software (Heinrich-Heine-Universität Düsseldorf) [34] based on a statistical test using repeated measures ANOVA. A type 1 error of .05 and a statistical power of 0.9 were used. The correlation among repeated measures was set conservatively at 0. Based on previous similar studies, an effect size of 0.25 was estimated. Based on these calculations, 22 participants were required. After estimating a 10% dropout rate, the required total sample size was set at 24 participants.

### Participants

Through flyer advertisements, 24 healthy males were recruited for this study from Chung-Ang University. The inclusion criteria were as follows: (1) male sex, (2) age 20 to 29 years, and (3) no psychiatric or medical illness. The exclusion criteria were as follows: (1) history of head trauma; (2) history of substance abuse, including alcohol, tobacco, and drugs; and (3) IQ<80. The participants were instructed not to consume any food, caffeine, or alcohol 3 hours prior to participation in the study.

### Ethics Approval

The institutional review board of Chung-Ang University approved this research protocol (1041078-201908-HRSB-231-01). Written informed consent was obtained from all participants.

### Study Procedure

#### Overview

**Figure 1:**
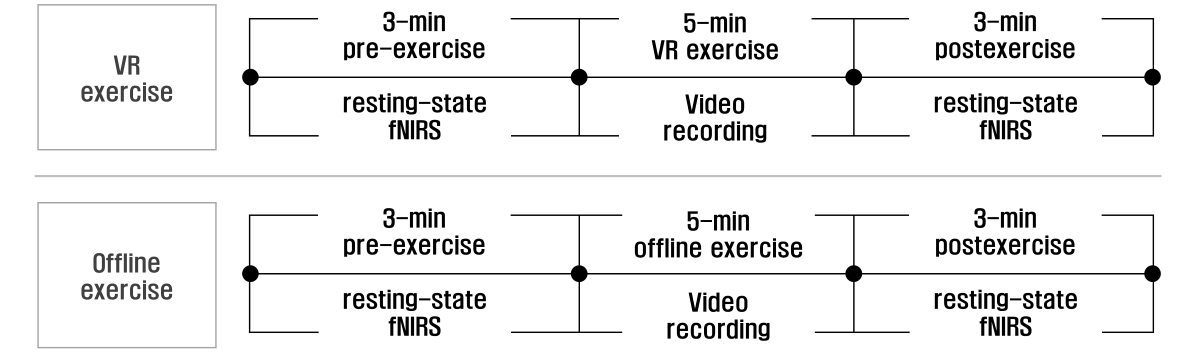
Study design. fNIRS: functional near-infrared spectroscopy; VR: virtual reality.

This study was a randomized crossover trial that compared responses to 3D immersive VR and offline exercises. The participants were blinded to the study hypotheses. Each participant underwent 1 3D immersive VR trial and 1 offline trial in a randomly assigned order at 10-minute intervals. Each session consisted of (1) a 2-minute practice to become familiarized with each exercise; (2) a 2-minute pre-exercise resting-state fNIRS measurement; (3) a 5-minute exercise, either 3D immersive VR or offline; and (4) a 3-minute postexercise resting-state fNIRS measurement (Figure 1). This trial was conducted in a curtained, dimly lit gym at a temperature of 23 °C with minimal ambient noise to control external noises as much as possible. The participants were instructed not to speak or move their heads during the fNIRS measurement to maintain good tissue-electrode contact. A 10-minute interval was given between sessions to return noncortical hemodynamic variables, such as skin blood flow and middle cerebral artery blood flow, to baseline levels.

Both VR and offline exercises consisted of 40 identical movements derived from Pilates. Pilates was chosen because it is a beneficial exercise method for healthy adults that enhances flexibility, muscular activity, and coordination [35]. In both exercises, the sequence was in the order of 8 types of simple behaviors involving just the body, arms, or legs; 16 types of 2 complex behaviors involving the body plus arms or legs; and 16 types of 3 complex behaviors involving 3 parts of the body, arms, and legs. The reason for exercising in this order was to gradually increase the number of muscle groups involved, starting from warming up. A resistance band (TheraBand) was applied to each participant in both sessions to maintain adequate exercise intensity and prevent less controlled behaviors. Pilates exercise with a resistance band was reported to increase muscle activity by more than 50%, whereas Pilates exercise without a resistance band increased muscle activity by only 20% [36].

Before the VR exercise, the research staff provided brief instructions on the 3D immersive VR system and environment and asked the participants to report whether they experienced any discomfort while wearing the HMD, such as head bumps, sweating, or motion sickness. The research staff checked for any obstacles the participants might encounter during the VR exercise and monitored and provided immediate assistance to the participants during the exercise in case of danger. With the help of the research staff, the participants were positioned 2 m horizontally away from the VR device, wearing the HMD and holding resistance bands in both hands at shoulder level.

**Figure 2:**
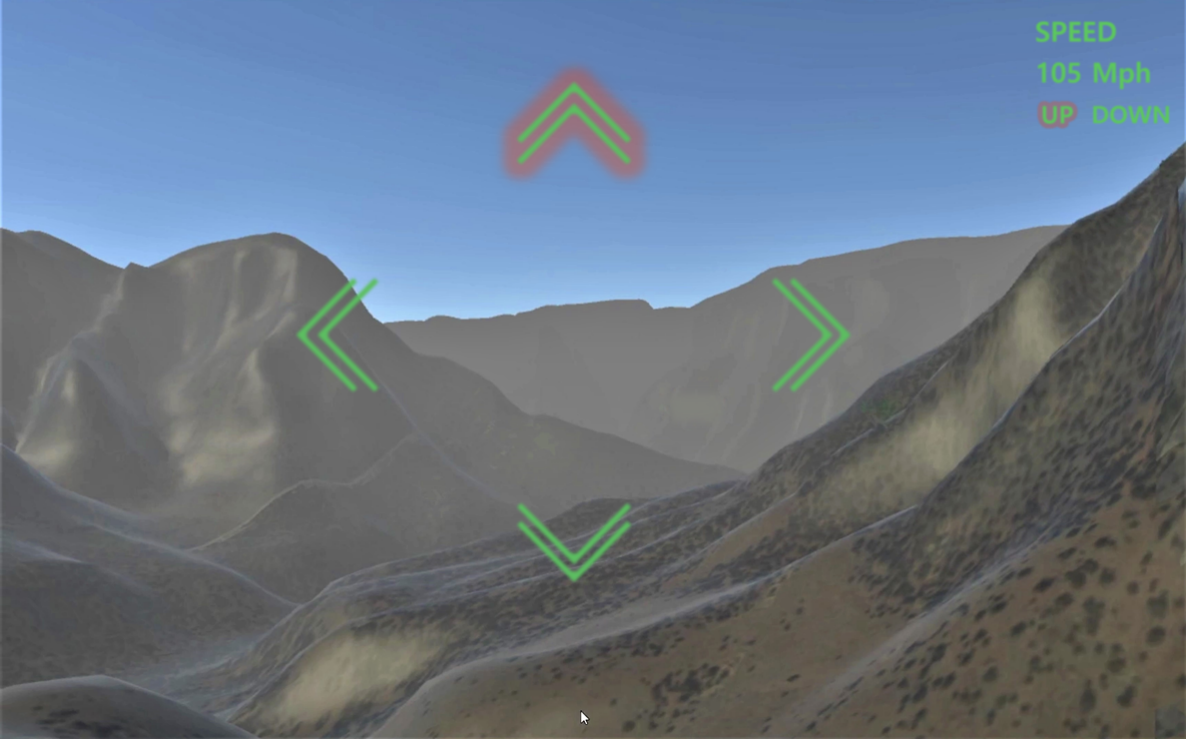
A representation of the virtual environment.

In this study, the Vive Cosmos Elite (High Tech Computer Corp) and Kinect V1 (Microsoft) were used as the HMD and motion sensors, respectively. A 3D map–based immersive VR software system that allows participants to interact with the VR environment was developed using Unity3D Engine (Unity Technologies). The Grand Canyon landscape was designed to enable participants to fly between canyons without crashing by performing the aforementioned movements in sequence. During the VR exercise, participants were asked to fly over the canyon without bumping into it, following visual and audio guidance. The direction in which to move, whether to accelerate or decelerate, and flight speed were displayed on the VR screen in real time, serving as visual instructions (Figure 2). When the participants tilted their upper body right, left, forward, or backward in the virtual space, they rotated right, left, upward, or downward, respectively. They could accelerate or decelerate by lifting or lowering their right arm.

In contrast, during the offline exercise, the participants performed movements in a standard manner following a demonstration by a professional instructor. In both sessions, audio guidance, such as “lean right and raise your left arm” were provided.

The research staff helped the participants accurately perform each movement in both sessions. They provided immediate feedback for incorrect movements and high-risk situations. The movements performed by the participants in the VR and offline conditions were considered identical since the audio guidance, use of the resistance band, and real-time feedback on behavior by the research staff were the same for all participants.

#### Motion Analysis to Measure Motion Angle, Length, and Retention Time

Participants’ movements during the VR and offline exercises were recorded using an iPhone 11 camera (Apple). The participants were asked to stand at the designated site 1.5 m away from the camera to maintain a constant camera angle. The recorded video was analyzed using the open-source video analysis software Kinovea (version 0.8.15; Kinovea Open-Source Project) [21]. Kinovea determines the distance between the camera and the recorded object by measuring the subject passing in front of the camera [21]. It has various analysis and measurement tools for adding annotations, drawing lines, and calculating distances and angles [37] and has been used in numerous sports and clinical studies [22-24]. Previous studies have shown the reliability and validity of the software at distances of up to 5 m with an angle of 90° to 45° [21].

**Figure 3:**
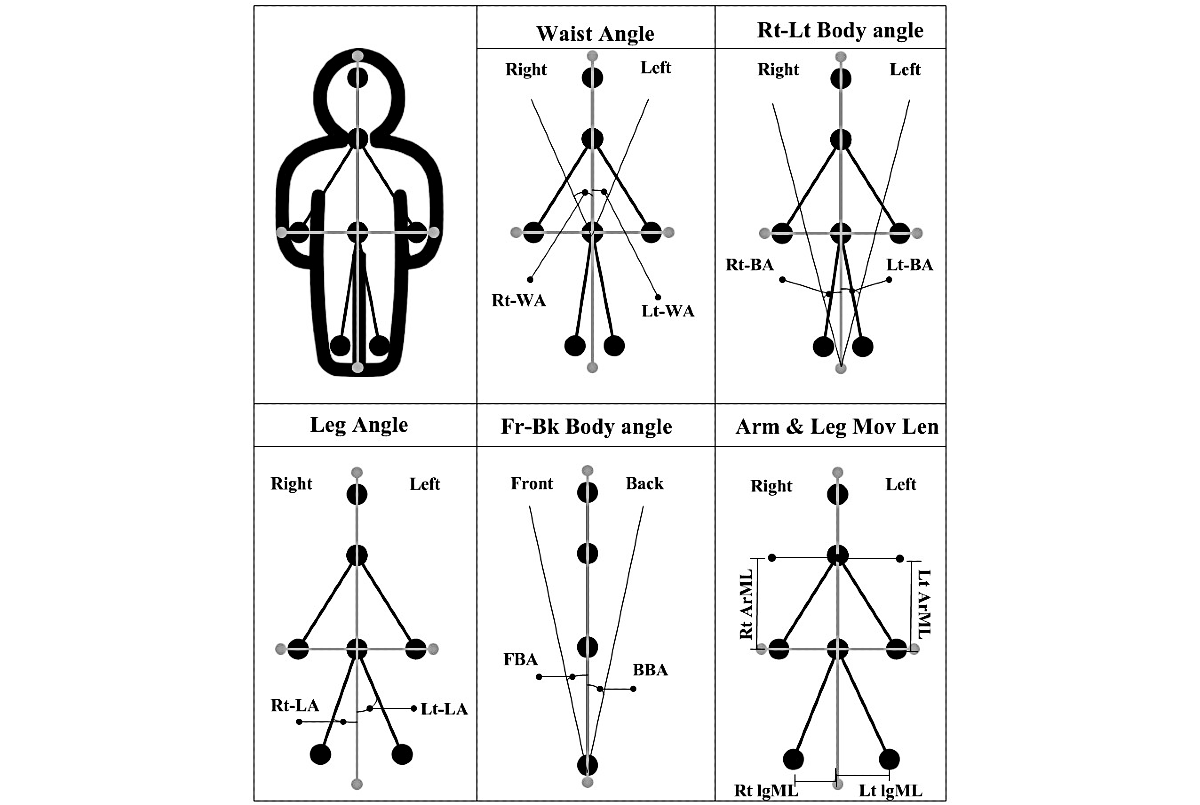
Movement angle and length. From top: Rt (Lt)-WA: right (left) waist angle; Rt (Lt)-BA: right (left) body angle; Rt (Lt)-LA: right (left) leg angle; FBA (BBA): forward (backward) body angle; Rt ArML: right arm movement length; Lt ArML: left arm movement length; Rt lgML: right leg movement length; Lt lgML: left leg movement length.

In the motion analysis, eight types of motion angles were measured: (1) right waist angle, (2) left waist angle, (3) right body angle, (4) left body angle, (5) right leg angle, (6) left leg angle, (7) forward body angle, and (8) backward body angle. Additionally, four types of movement lengths were measured: (1) right arm movement length, (2) left arm movement length, (3) right leg movement length, and (4) left leg movement length (Figure 3). The total angle and movement length for simple behaviors, 2 complex behaviors, and 3 complex behaviors were calculated using each angle and movement length involved in each behavior. For example, for simple behaviors, 8 angles and 4 lengths were used to calculate the total angle and movement lengths. Two complex behaviors consisting of 16 movements, 24 angles, and 16 lengths were measured to calculate the total angle and movement length. Finally, 3 complex behaviors consisting of 16 movements, 24 angles, and 32 lengths were measured to calculate the total angle and movement length (Multimedia Appendix 1).

#### Comparison of Calories Using the Metabolic Equivalent of Task Formula and Motion Retention Time

Calorie consumption during each session was calculated using each participant’s weights, motion retention time (from the start to the end of the movement), and the metabolic equivalent of task (MET) of the Pilates motion, according to the MET formula [38]. The MET formula has been adopted in various clinical studies, and its reliability has been demonstrated through meta-analyses [39,40]. MET is defined as the level of intensity of various physical activities, including daily activities and low-intensity to high-intensity exercises. The Pilates movements performed by the participants in this study were rated as 3.5 MET.

#### Hemodynamic Changes in the Frontal Cortex

Hemodynamic changes within the prefrontal cortex were assessed using a high-density fNIRS device (NIRSIT, OBELAB Inc). The curved panel of the NIRSIT has 24 laser diodes (sources) emitting 2 wavelengths (780 nm and 850 nm) of light and 32 photodetectors with a sampling rate of 8.138 Hz. The unit distance between the source and photodetectors was 15 mm. In this study, only the 30-mm channels were analyzed, since 30 mm is the most suitable sensor-detector separation distance for measuring cortical hemodynamic changes.

The fNIRS data were processed using the analysis toolbox provided by the fNIRS device manufacturer. The light signals in each wavelength within the 48 channels were filtered using a band-pass filter (between 0.0 Hz and 0.1 Hz) to reduce noise due to external light and body movements. Data derived from channels representing low-quality information (signal-to-noise ratio<30 dB) were not included in the hemodynamic analysis set to prevent misinterpretation. Relative hemodynamic changes during exercise were calculated using the modified Beer-Lambert law [41]. The accumulated oxygenated hemoglobin (accHbO2) values represent the activation of the prefrontal cortex during a resting state. Although both oxygenated and deoxygenated hemoglobin data could be obtained, previous studies have revealed that oxygenated hemoglobin has superior sensitivity and signal-to-noise ratio; therefore, only oxygenated hemoglobin data were used for this analysis [26,28].

**Figure 4:**
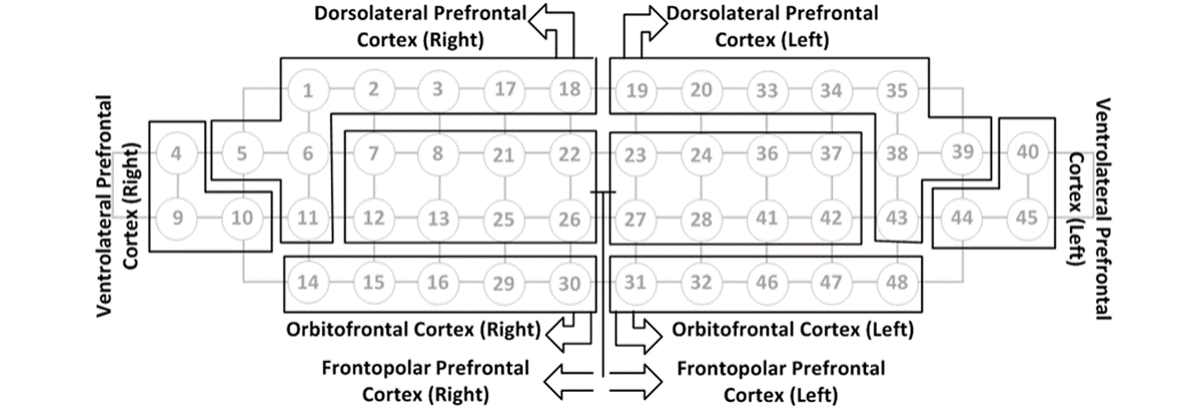
The channel configuration.

The values (mean and SD) of accHbO2 were gathered from 8 regions of interest: the right and left DLPFC, right and left frontopolar cortex (FPC), right and left VLPFC, and right and left OFC. The right and left DLPFCs were composed of channels 1, 2, 3, 5, 6, 11, 17, and 18 and channels 19, 20, 33, 34, 35, 38, 39, and 43, respectively. The right and left FPC were composed of channels 7, 8, 12, 13, 21, 22, 25, and 26 and channels 23, 24, 27, 28, 36, 37, 41, and 42, respectively. The right and left VLPFCs were composed of channels 4, 9, and 10 and channels 40, 44, and 45, respectively. The right and left OFCs were composed of channels 14, 15, 16, 29, and 30, and channels 31, 32, 46, 47, and 48, respectively (Figure 4).

### Statistical Analyses

For total behaviors, simple behavior, 2 complex behaviors, and 3 complex behaviors, the differences in total angles, total movement length, and consumed calories were compared using the Mann-Whitney *U* test. The statistical significance of the total angle and total movement length in the total behaviors was set at 0.02 (0.05/3, angle, length, and calories). In the post hoc test, the statistical significance of the total angle and total movement length in the simple behaviors, 2 complex behaviors, and 3 complex behaviors was set at *P*=.02 (.05/3, angle, length, and calories). Between the VR and offline situations, the difference in the change in accumulated oxygenated hemoglobin (ΔaccHbO2) from baseline and exercise was compared using repeated measures ANOVA. The statistical significance was set at *P*=.006 (.05/8, 8 brain regions). Correlations between the differences in accHbO2 within the right OFC, total angles, and total movement lengths were assessed using Pearson correlation coefficient. Statistical significance was set at *P*=.03 (.05/2, angle and length). Statistical analyses were performed using SPSS software (version 24.0; IBM Corp).

## Results

### Differences in Movement Including Angle and Length Between Offline and VR Trials During Exercise

During exercise, there were significant differences in the total angle (*z*=−2.31; *P*=.02) and length between the VR and offline trials (z=−2.78; *P*=.005). The VR group showed a 13.3% wider total angle range and an increase of 14.1% in the total movement length compared with the offline group. In the post hoc analysis, the VR group moved significantly more in simple and complex behaviors, except for the total angle in 2 complex behaviors, compared to the offline group (Table 1).

**Table 1:** Differences in angle and distance between the VR^a^ and offline trials^b^.

Types of behavior and parameters		VR trial, mean (SD)		Offline trial, mean (SD)		Statistics
**Total behavior**						
	Angle total (°)	1359.39 (125.55)		1200.26 (195.68)		*z*=−2.31; *P*=.02
	Length total (cm)	2568.20 (342.38)		2250.82 (382.20)		*z*=−2.78; *P*=.005
	Calorie total (kcal)	9.36 (2.60)		5.79 (3.01)		*z*=−3.04; *P*=.002
**Simple behavior (trunk leaning over)**						
	Angle total (°)	172.30 (19.2)		147.8 (27.64)		*z*=−2.74; *P*=.005
	Length total (cm)	224.93 (34.3)		197.70 (31.9)		*z*=−2.53; *P*=.01
	Calorie total (kcal)	2.46 (0.55)		1.18 (0.69)		*z*=−4.30; *P*<.001
**2 complex behaviors (trunk leaning over + lifting arm or leg)**						
	Angle total (°)	449.26 (118.57)	415.87 (69.17)		*z*=−1.14; *P*=.27	
	Length total (cm)	966.09 (118.57)	860.06 (107.01)		*z*=−3.10; *P*=.001	
	Calorie total (kcal)	4.42 (1.35)	2.95 (1.58)		*z*=−2.55; *P*=.01	
**3 complex behaviors (trunk leaning over + lifting arm + lifting leg)**						
	Angle total (°)	737 .83 (63.61)	636.55 (112.77)		*z*=−2.72; *P*=.006	
	Length total (cm)	1377.18 (202.88)	1193.05 (125.55)		*z*=−2.69; *P*=*.*006	
	Calorie total (kcal)	2.47 (0.86)	1.65 (0.97)		*z*=−2.63; *P*=.009	

^a^VR: virtual reality.

^b^*P*<.02 was considered to be significant.

### Differences in Consumption Between Offline and VR Trials During Exercise

During exercise, there were significant differences in the total calories between the VR and offline trials (*z*=−3.04; *P*=*.*002). During exercise, the VR group consumed 61.7% more calories than the offline group. In the post hoc analysis, the VR group showed significant calorie consumption with 2 complex and 3 complex behaviors compared to the offline group (Table 1).

### Differences in ΔaccHbO2 Between Offline and VR Trials During Exercise

**Figure 5:**
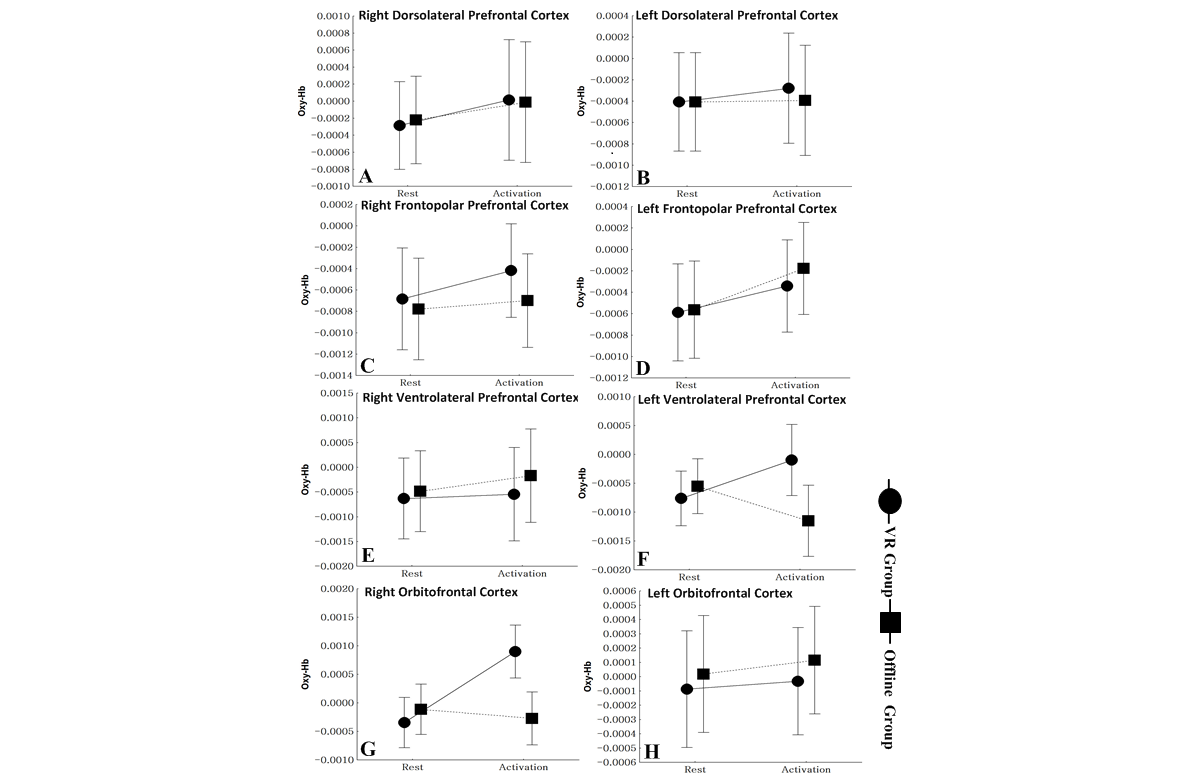
Difference in the accumulated oxygenated hemoglobin (ΔaccHbO2) between the offline and virtual reality trials during exercise. *P*<.006 was considered to be significant. Oxy-Hb: oxygenated hemoglobin; VR: virtual reality.

During exercise, significant differences were observed in ΔaccHbO2 between the offline and VR trials within the right OFC (*F*^1,94^=9.36; *P=.*003) but not within the left VLPFC (*F*^1,94^=5.69; *P=.*02), left DLPFC (*F*^1,94^=0.06; *P=.*81), right DLPFC (*F*^1,94^=0.02; *P=.*89), right VLPFC (*F*^1,94^=0.06; *P=.*81), left OFC (*F*^1,94^=0.01; *P=.*91), left frontopolar prefrontal cortex (*F*^1,94^=0.05; *P=.*89), or right frontopolar prefrontal cortex (*F*^1,94^=0.21; *P=.*65) (Figure 5).

### Correlation of ΔaccHbO2 With Total Angle and Movement Length

**Figure 6:**
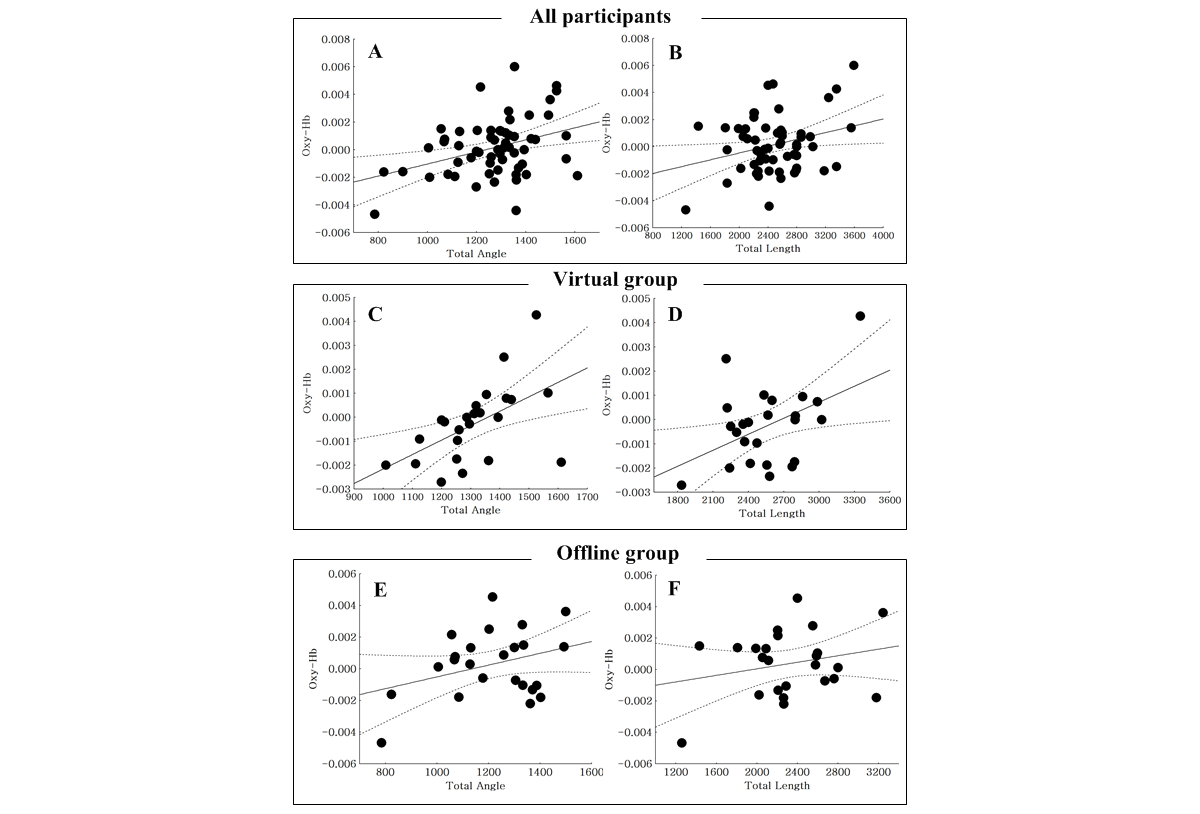
Correlation between accumulated oxygenated hemoglobin in the orbitofrontal cortex (OFC) and movement change. *P*<.025 was considered to be significant. Oxy-Hb: oxygenated hemoglobin.

In all participants, the ΔaccHbO2 within the right OFC was positively correlated with the total angle (*r*=0.36; *P*=.005). However, it was not significantly associated with the total movement length (*r*=0.28; *P*=.03). In the VR group, the ΔaccHbO2 within the right OFC was positively correlated with total angle (*r*=0.45; *P*=.001) and total movement length (*r*=0.38; *P*=.007). In the offline group, the ΔaccHbO2 was not significantly correlated with total angle (*r*=0.28; *P*=.40) or total movement length (*r*=0.29; *P*=.37) (Figure 6).

### User Experience During VR Exercise

Of the 24 participants, 3 (12.5%) reported motion sickness, and 8 (33.3%) reported sweating after wearing the HMD device; however, none reported HMD slipping or severe discomfort.

## Discussion

### Principal Results

In this study, VR exercise increased the range of motion, calorie consumption, and brain activity within the OFC compared with offline exercise. In addition, increased brain activity within the right OFC was correlated with an increased range of motion.

### Improvement of Movement Range and Calorie Consumption in Response to Immersive VR Stimuli

Our findings are consistent with previous studies on various patient groups. However, no studies have reported changes in the range of motion due to VR exercise in healthy individuals, as in this study [5]. VR exercises have been suggested to improve the range of motion in patients undergoing rehabilitation, both in the short term [42,43] and in the long term [14,44]. Immersive VR physical therapy significantly reduces pain and enhances the range of motion in patients with burn injuries (immediate effect) or chronic frozen shoulders (chronic effect) compared with standard therapies [14,42]. In addition, a 4-week VR exercise program in patients who underwent a stroke was more effective than a conventional program in improving the active range of motion and other scores, such as the Fugl-Meyer assessment score, Wolf motor function test score, and modified Barthel Index [44]. Children with cerebral palsy also reported a greater range of motion, better motion control, and greater interest as an acute effect after performing VR exercises than after conventional exercises [43]. In addition, the results of this study are in line with those of previous studies showing that participants who engaged in VR exercises had higher heart rates and burned more calories than those who performed standard exercises [45]. Therefore, VR has the potential to enhance physical exercises, and it may be a viable complement or alternative to traditional weight loss programs [46].

The larger range of motion and caloric consumption in the VR exercise may be associated with immersion. Immersion can be divided into 3 forms: sensory, imaginative, and challenge-based [47]. Sensory immersion is associated with audiovisual stimulation, and imaginative immersion is related to absorption in the narrative or identification with a character (eg, feelings of empathy and atmosphere). Challenge-based immersion is related to the balance between the activity’s demands and an individual’s motor and mental skills [48]. Challenge-based immersion is very close to the “flow” experience described by Csikszentmihalyi [49]. Immersion and flow share common features such as concentration, distortion of time perception, loss of self-awareness, and intrinsic motivation toward an activity [49]. The flow of physical exercise is related to peak performance [50]. The flow experienced during exercise is an autotelic experience with total concentration, merging of action and awareness, and the paradox of control [51]. In this respect, we cautiously suggest that VR exercise could be more immersive and helpful in achieving peak performance than exercise alone since sensory and imaginative components are added to the challenge-based immersion of physical exercise.

### Increased Brain Activity Within OFC in Response to VR Stimuli

Another important finding of our study was that there were significant differences in brain activation within the right OFC between offline and exercise groups. To date, only a few studies have specifically investigated orbitofrontal activity in response to VR exercises. Most studies have focused on prefrontal activation caused by VR stimuli. Mao et al [52] suggested that VR training activates the prefrontal cortex and improves spatial orientation and motor function. However, recent studies have suggested that the OFC is one of the candidate brain regions that respond to VR [29]. Landowska et al [21] reported hemodynamic changes within the DLPFC and OFC in response to VR therapy for acrophobia. Dong et al [53] suggested that the activation of the OFC in response to VR tasks was greater than that observed in response to slide-based (control) tasks. Moro et al [54] reported that oxygenation in the OFC was increased when an incremental swing balance task in an immersive VR environment was performed. One possible explanation for this is that VR-induced immersion stimulates reward circuits involving the OFC. The OFC is responsible for sensory integration, regulation of visceral responses, learning, prediction, and decision-making for reward and affective values [55,56]. In addition, visual stimulation and immersion in VR are robustly involved in reward circuits [57].

The results of the correlation study between ΔaccHbO2 within the right OFC and the range of motion during VR and offline exercise showed that an increase in the range of motion and right OFC activation was correlated only in the VR group but not in the offline group. Thus, we suggest that immersion and visual stimulation of 3D immersive VR distracts the user from unpleasant interoceptive sensations caused by physical exercise. It is suggested that distraction caused by VR can reduce the perception of physical discomfort through an intercortical modulation of the anterior cingulate and OFC [9,58,59].

Additionally, the functional hemispheric differences shown in the results of this study are in line with a previous study that suggested that the more pronounced the shift in brain activity to the right hemisphere, the more flow experienced by elite tennis players [60]. Less synchronized brain activity in the left hemisphere and more coherent brain activity in the right hemisphere have been suggested to reflect less interference from irrelevant verbal-analytical processes with motor control mechanisms [60].

### Strengths

To the best of our knowledge, this is one of the earliest studies to quantitatively evaluate the effects of 3D immersive VR exercise on brain hemodynamics in healthy individuals. Each participant’s movement was measured using professional video analysis software, unlike other studies based on subjective performance improvement or satisfaction. Another strength of this study is that we directly compared performance in 3D immersive VR and real-world environments while performing the same activities. This comparison is important to reveal how 3D immersive VR influences exercise performance and brain activity. Finally, this study presents the potential for developing smart headsets by integrating wearable devices and connected technologies, such as VR, brain activity measurement, and motion tracking. Although these were performed with different devices in our study, using an integrated smart headset could enable effective exercise and immediate checking of the exercise amount and the consequent changes in brain activity. These wearable technologies and connected solutions with VR capabilities are already widely adopted in the workplace to promote occupational safety, productivity, and worker health. Examples include the Xsens MVN Animate and Xsens MTw Awinda for body movement tracking and the Muse 2 Melon's headband and IMEC's wireless electroencephalogram headset for brain wave sensing [61].

### Limitations

This study had some limitations. First, due to the small number and gender disparity of the participants, the results cannot be generalized. In a follow-up study on VR exercise, scaling up the sample size by including virtual participants could be considered. However, this was not possible in this study due to the crossover design comparing VR and offline exercise. Second, there were limitations in the adequate pixel rate, capture rate, maximum aperture, depth of field capture, and frame capture rate of the iPhone 11, which was used as a video recording device. Third, since the fNIRS device used in this study can only measure brain activity within the prefrontal lobe, activity in other cortical regions and deep brain areas was not measured. Further studies using other functional imaging devices, such as fMRI, are required to determine the activity of the entire brain. Fourth, we did not employ additional physiological measures. The use of systemic physiologically augmented fNIRS in future studies may yield more meaningful results. Finally, a standardized questionnaire to check the inclusion and exclusion criteria and to assess motion sickness after VR exercise was not used in this study.

### Conclusions

This study confirmed that 3D immersive VR exercise is more effective at increasing the range of motion and calorie consumption during exercise in healthy individuals via activation within the OFC.

## Appendix

Multimedia Appendix 1 Performances for simple and complex behaviors.

Multimedia Appendix 2 CONSORT-eHEALTH checklist (V 1.6.2).

## Abbreviations

accHbO2: accumulated oxygenated hemoglobin
DLPFC: dorsolateral prefrontal cortex
fMRI: functional magnetic resonance imaging
fNIRS: functional near-infrared spectroscopy
FPC: frontopolar cortex
HMD: head-mounted display
MET: metabolic equivalent of task
OFC: orbitofrontal cortex
VLPFC: ventrolateral prefrontal cortex
VR: virtual reality
ΔaccHbO2: change in accumulated oxygenated hemoglobin
