# Supplementary material for: Differences in Brain Activity and Body Movements Between Virtual Reality and Offline Exercise: Randomized Crossover Trial
Source: JMIR Serious Games. 2023 Jan 5;11:e40421. doi: 10.2196/40421 (PMC9853339; doi:10.2196/40421)
Supplement: Multimedia Appendix 1 [file games_v11i1e40421_app1.docx]

Multimedia Appendix 1: Performances in simple and complex behaviors.

| Body | Arm | Leg |
| --- | --- | --- |
| **Simple behavior** | | |
| leaning left^a^ |  |  |
| leaning right^a^ |  |  |
| leaning forward^b^ |  |  |
| leaning backward^b^ |  |  |
|  | lifting left arm |  |
|  | lifting right arm |  |
|  |  | lifting left leg |
|  |  | lifting right leg |
| **2 complex behavior** | | |
| leaning left/right^c^ | lifting left arm |  |
|  | lifting right arm |  |
| leaning forward/backward^d^ | lifting left arm |  |
|  | lifting right arm |  |
| leaning left/right^e^ |  | lifting left leg |
|  |  | lifting right leg |
| leaning forward/backward^f^ |  | lifting left leg |
|  |  | lifting right leg |
| **3 complex behavior** | | |
| leaning left/right^g^ | lifting left arm | lifting left leg (2 angle, 2 length) |
|  |  | lifting right leg (2 angle, 2 length) |
|  | lifting right arm | lifting left leg (2 angle, 2 length) |
|  |  | lifting right leg (2 angle, 2 length) |
| leaning forward/ backward^h^ | lifting left arm | lifting left leg (1 angle, 2 length) |
|  |  | lifting left leg (1 angle, 2 length) |
|  | lifting right arm | lifting right leg (1 angle, 2 length) |
|  |  | lifting right leg (1 angle, 2 length) |

^a^ 2 angles = waist and body

^b^ one body angle

^c^ 8 angles = 2 (left and right leaning) × 2 angles (waist and body) × 2 (left and right arm), 4 lengths = 2 (left and right leaning) × 2 (left and right arm lifting)

^d^ 4 angles = 2 (forward and backward) × 2 body, 4 lengths = 2 (forward and backward) × 2 (left and right arm lifting)

^e^ 8 angles = 2 (left and right leaning) × 2 (waist and body) × 2 (left and right leg lifting), 4 lengths = 2 (forward and backward) × 2 (left and right leg lifting)

^f^ 4 angles = 2 (forward and backward) × 2 body, 4 lengths = 2 (forward and backward) × 2 (left and right left lifting)

^g^ 16 angles = 2 (left and right leaning) × 2 angles (waist and body) × 2 (left and right arm lifting) × 2 (left and right leg lifting), 16 lengths = 2 (left and right leaning) × 2 (left and right arm lifting) × 2 (left and right leg lifting) × 2 (arm and leg cross)

^h^ 8 angles = 2 (left and right leaning) × 1 body angle × 2 (left and right arm lifting) × 2 (left and right leg lifting), 16 lengths = 2 (forward and backward) × 2 (left and right arm lifting) × 2 (left and right leg lifting) × 2 (arm and leg crossing)
