# Supplementary material for: Differences in Brain Activity and Body Movements Between Virtual Reality and Offline Exercise: Randomized Crossover Trial
Source: JMIR Serious Games. 2023 Jan 5;11:e40421. doi: 10.2196/40421 (PMC9853339; doi:10.2196/40421)
Supplement: Multimedia Appendix 2 [file games_v11i1e40421_app2.pdf]

CONSORT-EHEALTH Checklist V1.6.2 Report	Manuscript Number	40421

(based on CONSORT-EHEALTH V1.6), available at [http://tinyurl.com/consort-ehealth-v1-6].

Date completed

12/20/2022 1:19:35

by

Hee Jin

TITLE

1a-i) Identify the mode of delivery in the title

1a-ii) Non-web-based components or important co-interventions in title

"Virtual Reality and Offline Exercise"

1a-iii) Primary condition or target group in the title

"Virtual Reality and Offline Exercise"

ABSTRACT

1b-i) Key features/functionalities/components of the intervention and comparator in the METHODS section of the ABSTRACT

1b-ii) Level of human involvement in the METHODS section of the ABSTRACT

"3D immersive virtual reality exercise"

1b-iii) Open vs. closed, web-based (self-assessment) vs. face-to-face assessments in the METHODS section of the ABSTRACT

The research staff provided instructions on the VR system and helpled the participants accurately perform each movements in both sessions. During offline session, professional instructor gave demonstrations in front of the participants. However, this was not addressed in the abstract due to the limited word count.

1b-iv) RESULTS section in abstract must contain use data

"Twenty-four healthy males performed the same motions when exercising with and without 3D immersive virtual reality, and the recorded videos were used for motion analysis. Hemodynamic changes in the prefrontal cortex were assessed using functional near-infrared spectroscopy." indicates that this trial was held face-to-face, and the outcomes were assessed by researchers.

1b-v) CONCLUSIONS/DISCUSSION in abstract for negative trials

The number of participants enrolled/assessed, the use of the intervention are addressed in Methods section in abstract as " Twenty-four healthy males performed the same motions when exercising with and without 3D immersive virtual reality".

INTRODUCTION

2a-i) Problem and the type of system/solution

2a-ii) Scientific background, rationale: What is known about the (type of) system

"This study aimed to investigate whether immersive VR technology can enhance physical exercises, regarding the range of motion and brain activity, in healthy individuals."

The 3D immersive VR program developed in this study was intended as stand-alone invervention, but it could also be incorporated in broader health care program, since it complements standard offline exercise.

METHODS

3a) CONSORT: Description of trial design (such as parallel, factorial) including allocation ratio

3b) CONSORT: Important changes to methods after trial commencement (such as eligibility criteria), with reasons

We hypothesized that 3D immersive VR exercises would be more effective than offline physical exercises in increasing the range of motion and calorie consumption during exercise. In addition, we hypothesized that the effectiveness of 3D immersive VR exercises are associated with brain activation within the OFC, before and after exercise.

3b-i) Bug fixes, Downtimes, Content Changes

4a) CONSORT: Eligibility criteria for participants

As our trial as randomized crossover trial, allocation ratio is not applicable/relevant.

"This study was a randomized crossover trial that compared responses to 3D immersive VR and offline exercises. The participants were blinded to the study hypotheses. Each participant underwent one 3D immersive VR trial and one offline trial in a randomly assigned order at ten-minute intervals."

4a-i) Computer / Internet literacy

4a-ii) Open vs. closed, web-based vs. face-to-face assessments:

4a-iii) Information giving during recruitment

"Through flyer advertisements, 24 healthy males were recruited for the current study at Chung-Ang University."

"This trial was conducted in a curtained, dimly lit gym at a temperature of 23°C with minimal ambient noise to control external noises as much as possible. The participants were instructed not to speak or move their heads during the fNIRS measurement to maintain good tissue-electrode contact." indicates that this trial was held face-to-face, and the outcomes were assessed by researchers.

4b) CONSORT: Settings and locations where the data were collected

No important changes to methods was made after trial commencement.

4b-i) Report if outcomes were (self-)assessed through online questionnaires

4b-ii) Report how institutional affiliations are displayed

"Participants' movements during the VR and offline exercises were recorded using an iPhone 11 camera (Apple, Mountain View, CA, USA)."

"Hemodynamic changes within the prefrontal cortex were assessed using a high-density fNIRS device (NIRSIT, OBELAB Inc., Seoul, Korea)."

5) CONSORT: Describe the interventions for each group with sufficient details to allow replication, including how and when they were actually administered

5-i) Mention names, credential, affiliations of the developers, sponsors, and owners

5-ii) Describe the history/development process

5-iii) Revisions and updating

5-iv) Quality assurance methods

5-v) Ensure replicability by publishing the source code, and/or providing screenshots/screen-capture video, and/or providing flowcharts of the algorithms used

5-vi) Digital preservation

5-vii) Access

5-viii) Mode of delivery, features/functionalities/components of the intervention and comparator, and the theoretical framework

This item is not very relevant to our study. Participants accessed the VR software "Grand Canyon", which was pre-installed on the VR device prepared for the experiment.		
**5-ix) Describe use parameters**		
"Moreover, VR exercises have recently been widely used by the general public to promote physical fitness [5]. When applied to healthy individuals, VR exercises increases exercise speed, heart rate, balance, motivation, enjoyment, adherence, executive function, and neuroplasticity [17-20]. Although many studies have suggested the qualitative effectiveness of VR exercise, few have quantitatively assessed its effectiveness [12].<br>Previous studies have suggested that the brain regions of interest in response to VR and physical exercises are the dorsolateral prefrontal cortex (DLPFC), ventrolateral prefrontal cortex (VLPFC), and orbitofrontal cortex (OFC) [9, 21, 22]. Of these three major prefrontal cortical regions, a functional near-infrared spectroscopy (fNIRS) study showed that VR stimuli activated the OFC [21]. The OFC has been associated with emotional and behavioral regulation, decision-making, maintenance of behavioral flexibility, and processing of anticipated rewards and punishments [23]. Inappropriate functioning of the OFC can lead to disinhibition, preservation, and impulse control problems [24]. Owing to the functions of rewards and decisions, the OFC can be associated with VR-induced immersion and flow [8, 25].<br>This study aimed to investigate whether immersive VR technology can enhance physical exercises, regarding the range of motion and brain activity, in healthy individuals."<br>"This study was a randomized crossover trial that compared responses to 3D immersive VR and offline exercises. The participants were blinded to the study hypotheses. Each participant underwent one 3D immersive VR trial and one offline trial in a randomly assigned order at ten-minute intervals. Each session consisted of 1) two-minute practice to become familiarized with each exercise; 2) pre-exercise three-minute resting-state fNIRS measurement; 3) five-minute exercise, either 3D immersive VR or offline; and 4) post-exercise three-minute resting-state fNIRS measurement (Figure 1)."<br>"Both VR and offline exercises consisted of 40 identical movements derived from Pilates."<br>"In both exercises, the sequence was in the order of eight types of simple behaviors involving just the body, arms, or legs; 16 types of two-complex behaviors involving the body + arms or legs; and 16 types of three-complex behaviors involving the three parts of the body, arms, and legs. The reason for exercising in this order was to gradually increase the number of muscle groups involved, starting from warming up. A TheraBand was applied to each participant in both sessions to maintain adequate exercise intensity and prevent less controlled behaviors."<br>"The Grand Canyon was designed such that participants could fly between canyons without crashing simply by performing the aforementioned movements in sequence. During the VR exercise, participants were asked to fly over the canyon without bumping into it, following visual and audio guidance. The direction in which to move, whether to accelerate or decelerate and flight speed were displayed on the VR screen in real-time, serving as visual instructions (Figure 2). When the participants tilted their upper body right, left, forward, or backward in the virtual space, they rotated right, left, upward, or downward, respectively. They could accelerate or decelerate by lifting or lowering their right arm.<br>In contrast, during the offline exercise, the participants performed movements in a standard manner following a professional instructor demonstration in front of them. In both sessions, audio guidance, such as “Lean right and Raise your left arm.” were provided."<br>"The movements performed by the participants in the VR and offline conditions were considered to be identical since the audio guidance, use of the TheraBand, and real-time feedback on behavior by the research staffs were the same for all participants."		
**5-x) Clarify the level of human involvement**		
**5-xi) Report any prompts/reminders used**		
**5-xii) Describe any co-interventions (incl. training/support)**		
Since our study was a one-time (not routine) randomized crossover study conducted face-to-face, no reminder was needed.		
**6a) CONSORT: Completely defined pre-specified primary and secondary outcome measures, including how and when they were assessed**		
The inclusion criteria were as follows:1) male sex; 2) age 20–29 years; and 3) no psychiatric or medical illness. The exclusion criteria were as follows:1) history of head trauma; 2) history of substance abuse, including alcohol, tobacco, and drugs; and 3) intelligence quotient (IQ) < 80.		
**6a-i) Online questionnaires: describe if they were validated for online use and apply CHERRIES items to describe how the questionnaires were designed/deployed**		
**6a-ii) Describe whether and how “use” (including intensity of use/dosage) was defined/measured/monitored**		
**6a-iii) Describe whether, how, and when qualitative feedback from participants was obtained**		
**6b) CONSORT: Any changes to trial outcomes after the trial commenced, with reasons**		
"This trial was conducted in a curtained, dimly lit gym at a temperature of 23°C with minimal ambient noise to control external noises as much as possible."		
**7a) CONSORT: How sample size was determined**		
**7a-i) Describe whether and how expected attrition was taken into account when calculating the sample size**		
**7b) CONSORT: When applicable, explanation of any interim analyses and stopping guidelines**		
"the differences in total angles, total movement length, and consumed calories"<br>"the difference in the change in accumulated oxygenated hemoglobin (ΔaccHbO2) from baseline and exercise"		
**8a) CONSORT: Method used to generate the random allocation sequence**		
No changes were made to the outcome after trail commenced.		
**8b) CONSORT: Type of randomisation; details of any restriction (such as blocking and block size)**		
Interim analysis was not planned or performed in our study.		
**9) CONSORT: Mechanism used to implement the random allocation sequence (such as sequentially numbered containers), describing any steps taken to conceal the sequence until interventions were assigned**		
Simple randomization via random number generator using Microsoft Excel® was used.		
**10) CONSORT: Who generated the random allocation sequence, who enrolled participants, and who assigned participants to interventions**		
Simple randomization via random number generator using Microsoft Excel® was used.		
**11a) CONSORT: Blinding - If done, who was blinded after assignment to interventions (for example, participants, care providers, those assessing outcomes) and how**		
**11a-i) Specify who was blinded, and who wasn’t**		
**11a-ii) Discuss e.g., whether participants knew which intervention was the “intervention of interest” and which one was the “comparator”**		
Neither the participants nor the researchers were blinded. However, the participants were blinded to the specific study hypotheses.<br>"The participants were blinded to the study hypotheses."		
**11b) CONSORT: If relevant, description of the similarity of interventions**		
Simple randomization via random number generator using Microsoft Excel® was used.		
**12a) CONSORT: Statistical methods used to compare groups for primary and secondary outcomes**		
The trial protocol can be accessed from the Clinical Research Information Service (KCT0008021).		
**12a-i) Imputation techniques to deal with attrition / missing values**		
**12b) CONSORT: Methods for additional analyses, such as subgroup analyses and adjusted analyses**		

"Both VR and offline exercises consisted of 40 identical movements derived from Pilates."
"In both exercises, the sequence was in the order of eight types of simple behaviors involving just the body, arms, or legs; 16 types of two-complex behaviors involving the body + arms or legs; and 16 types of three-complex behaviors involving the three parts of the body, arms, and legs. The reason for exercising in this order was to gradually increase the number of muscle groups involved, starting from warming up. A TheraBand was applied to each participant in both sessions to maintain adequate exercise intensity and prevent less controlled behaviors."
"The Grand Canyon was designed such that participants could fly between canyons without crashing simply by performing the aforementioned movements in sequence. During the VR exercise, participants were asked to fly over the canyon without bumping into it, following visual and audio guidance. The direction in which to move, whether to accelerate or decelerate and flight speed were displayed on the VR screen in real-time, serving as visual instructions (Figure 2). When the participants tilted their upper body right, left, forward, or backward in the virtual space, they rotated right, left, upward, or downward, respectively. They could accelerate or decelerate by lifting or lowering their right arm.
In contrast, during the offline exercise, the participants performed movements in a standard manner following a professional instructor demonstration in front of them. In both sessions, audio guidance, such as "Lean right and Raise your left arm." were provided."
"The movements performed by the participants in the VR and offline conditions were considered to be identical since the audio guidance, use of the TheraBand, and real-time feedback on behavior by the research staffs were the same for all participants."

RESULTS

13a) CONSORT: For each group, the numbers of participants who were randomly assigned, received intended treatment, and were analysed for the primary outcome

13b) CONSORT: For each group, losses and exclusions after randomisation, together with reasons

No other analyses were performed.

13b-i) Attrition diagram

14a) CONSORT: Dates defining the periods of recruitment and follow-up

"Each participant underwent one 3D immersive VR trial and one offline trial in a randomly assigned order at ten-minute intervals." indicates that all 24 participants underwent randomization. Twelve participants conducted the VR session first, followed by the offline session, and the other 12 participants conducted the offline session first, followed by the VR session.

14a-i) Indicate if critical "secular events" fell into the study period

14b) CONSORT: Why the trial ended or was stopped (early)

There were no loss and exclusions after randomization.

15) CONSORT: A table showing baseline demographic and clinical characteristics for each group

Participants were recruited through flyer advertisements between May 2020 and June 2020, and the experiment was conducted on June 24th, 2020 (one single day experiment).

15-i) Report demographics associated with digital divide issues

16a) CONSORT: For each group, number of participants (denominator) included in each analysis and whether the analysis was by original assigned groups

16-i) Report multiple "denominators" and provide definitions

16-ii) Primary analysis should be intent-to-treat

"Through flyer advertisements, 24 healthy males were recruited for the current study at Chung-Ang University. The inclusion criteria were as follows:1) male sex; 2) age 20–29 years; and 3) no psychiatric or medical illness. The exclusion criteria were as follows:1) history of head trauma; 2) history of substance abuse, including alcohol, tobacco, and drugs; and 3) intelligence quotient (IQ) < 80. The participants were informed not to consume any food, caffeine, or alcohol three hours prior to participation in the study. Written informed consent was obtained from all participants."
Since this was a randomized crossover trial, all 24 participants underwent one 3D immersive VR trial and one offline trial in a randomly assigned order at ten-minute intervals.

17a) CONSORT: For each primary and secondary outcome, results for each group, and the estimated effect size and its precision (such as 95% confidence interval)

The trial was not stopped or ended early.

17a-i) Presentation of process outcomes such as metrics of use and intensity of use

17b) CONSORT: For binary outcomes, presentation of both absolute and relative effect sizes is recommended

As the participants were 24 healthy males who met the following inclusion and exclusion criteria, we decided that baseline demographic and clinical characteristics were not necessary.
"The inclusion criteria were as follows:1) male sex; 2) age 20–29 years; and 3) no psychiatric or medical illness. The exclusion criteria were as follows:1) history of head trauma; 2) history of substance abuse, including alcohol, tobacco, and drugs; and 3) intelligence quotient (IQ) < 80."

18) CONSORT: Results of any other analyses performed, including subgroup analyses and adjusted analyses, distinguishing pre-specified from exploratory

"During exercise, there were significant differences in the total angle (z=-2.31, P=.015) and length between the VR and offline trials (z=-2.78, P=.005). The VR group showed a 13.3% wider total angle range and an increase of 14.1% in the total movement length compared with the offline group."
"During exercise, there were significant differences in the total calories between the VR and offline trials (z=-3.04, P=.002). During exercise, the VR group consumed 38.0% more calories than the offline group."
"During exercise, significant differences were observed in the changes in accHbO2 (ΔaccHbO2) between the offline and VR trials within the right OFC (F=9. 36, P=.003), but not within the left VLPFC (F=5.69, P=.02), left DLPFC (F=0.06, P=.81), right DLPFC (F=0.02, P=.89), right VLPFC (F=0.06, P=.81), left OFC (F=0.01, P=.91), left frontopolar prefrontal cortex (F=0.05, P=.89), or right frontopolar prefrontal cortex (F=0.21, P=.65) (Figure 5)."
"In all participants, the ΔaccHbO2 within the right OFC was positively correlated with the total angle (r=0.36, P=.005). However, it was not significantly associated with the total movement length (r=0.28, P=.03). In the VR group, the ΔaccHbO2 within the right OFC was positively correlated with total angle (r=0.45, P=.001) and total movement length (r=0.38, P=.007). In the offline group, the ΔaccHbO2 was not significantly correlated with total angle (r=0.28, P=.40) or total movement length (r=0.29, P=.37) (Figure 6)."

18-i) Subgroup analysis of comparing only users

19) CONSORT: All important harms or unintended effects in each group

Our trial did not use any binary outcomes. Therefore, this item is not applicable.

19-i) Include privacy breaches, technical problems

19-ii) Include qualitative feedback from participants or observations from staff/researchers

DISCUSSION

20) CONSORT: Trial limitations, addressing sources of potential bias, imprecision, multiplicity of analyses

20-i) Typical limitations in ehealth trials

21) CONSORT: Generalisability (external validity, applicability) of the trial findings

21-i) Generalizability to other populations

21-ii) Discuss if there were elements in the RCT that would be different in a routine application setting

22) CONSORT: Interpretation consistent with results, balancing benefits and harms, and considering other relevant evidence

22-i) Restate study questions and summarize the answers suggested by the data, starting with primary outcomes and process outcomes (use)

22-ii) Highlight unanswered new questions, suggest future research

"In this study, VR exercise increased the range of motion, calorie consumption, and brain activity within the OFC compared with offline exercise. In addition, increased brain activity within the right OFC was correlated with an increased range of motions."<br>"The larger range of motion and caloric consumption in the VR exercise may be associated with immersion."<br>"In this respect, we cautiously suggest that VR exercise would be more immersive and helpful in achieving peak performance than exercise alone since sensory and imaginative components are added to the challenge-based immersion of physical exercises."<br>"One possible explanation for this is that VR-induced immersion stimulates reward circuits involving the OFC. The OFC is responsible for sensory integration, regulation of visceral responses, learning, prediction, and decision-making for reward and affective values [56, 57]. In addition, visual stimulation and immersion in VR are robustly involved in reward circuits [58]."<br>"The results of the correlation study between ΔaccHbO2 within the right OFC and the range of motion during VR and offline exercise showed that an increase in range of motion and right OFC activation was correlated only in the VR group but not in the offline group. Thus, we suggest that immersion and visual stimulation of 3D immersive VR distracts the user from the unpleasant interoceptive sensations caused by physical exercises. Previous studies have also suggested that distraction caused by the VR environment can reduce the perception of physical discomfort by intercortical modulation of the signal pathways of the anterior cingulate and OFC [9, 59, 60]."		
Other information		
**23) CONSORT: Registration number and name of trial registry**		
No other analyses were performed.		
**24) CONSORT: Where the full trial protocol can be accessed, if available**		
"Of the 24 participants, three reported motion sickness, and eight reported sweating after wearing the HMD device; however, none reported the HMD slipping or severe discomfort."		
**25) CONSORT: Sources of funding and other support (such as supply of drugs), role of funders**		
This trial is registered in the Clinical Research Information Service (KCT0008021), a<br>member of the WHO International Clinical Trials Registry Platform.		
**X26-i) Comment on ethics committee approval**		
**x26-ii) Outline informed consent procedures**		
"The institutional review board of Chung-Ang University approved the research protocol (1041078-201908-HRSB-231-01)."		
**X26-iii) Safety and security procedures**		
"Written informed consent was obtained from all participants."		
**X27-i) State the relation of the study team towards the system being evaluated**		
